# Correction to: Evaluation of the clinical evolution and transmission of SARS-CoV-2 infection in cats by simulating natural routes of infection

**DOI:** 10.1007/s11259-022-09955-y

**Published:** 2022-06-14

**Authors:** Sandra Barroso-Arevalo, Lidia Sanchez-Morales, Jose A. Barasona, Belén Rivera, Rocío Sánchez, María A. Risalde, Irene Agullo-Ros, José M. Sanchez-Vizcaino

**Affiliations:** 1 VISAVET Health Surveillance Center, Complutense University of Madrid, Madrid, Spain; 2 Department of Animal Health, Faculty of Veterinary, Complutense University of Madrid, Madrid, Spain; 3 Research Group in Animal Health and Zoonoses (GISAZ), Department of Anatomy and Comparative Pathology, Faculty of Veterinary, University of Cordoba, Andalusia, Spain; 4 Infectious Diseases Unit, Clinical Virology and Zoonosis Group, Maimonides Biomedical Research Institute of Cordoba (IMIBIC), Reina Sofia University Hospital, Andalusia, Spain

**Correction to: Veterinary Research Communications**

10.1007/s11259-022-09908-5

Following publication of this article, the complete funding information was omitted. It should be “This research was funded by the Institute of Health Carlos III (ISCIII) with the project “Estudio del potencial impacto del COVID19 en mascotas y linces” (reference: COV20/01385) and also financed as part of the Union's response to the COVID-19 pandemic, being the financing entities the Community of Madrid and the European Union, through the European Regional Development Fund (ERDF) instead of "This research was funded by the Institute of Health Carlos III (ISCIII) with the project “Estudio del potencial impacto del COVID19 en mascotas y linces” (reference: COV20/01385).

The original article has been corrected.

